# Ultrasonic Velocity and Attenuation of Low-Carbon Steel at High Temperatures

**DOI:** 10.3390/ma16145123

**Published:** 2023-07-20

**Authors:** Jan Lean Tai, Mohamed Thariq Hameed Sultan, Andrzej Łukaszewicz, Farah Syazwani Shahar, Wojciech Tarasiuk, Jerzy Napiórkowski

**Affiliations:** 1 Department of Aerospace Engineering, Faculty of Engineering, Universiti Putra Malaysia, Serdang 43400, Selangor, Malaysia; taijanlean2008@hotmail.com (J.L.T.); farahsyazwani@upm.edu.my (F.S.S.); 2 Laboratory of Biocomposite Technology, Institute of Tropical Forest and Forest Product (INTROP), University Putra Malaysia, Serdang 43400, Selangor, Malaysia; 3 Aerospace Malaysia Innovation Centre [944751-A], Prime Minister’s Department, MIGHT Partnership Hub, Jalan Impact, Cyberjaya 63600, Selangor, Malaysia; 4 Institute of Mechanical Engineering, Faculty of Mechanical Engineering, Bialystok University of Technology, 15-351 Bialystok, Poland; w.tarasiuk@pb.edu.pl; 5 Faculty of Technical Sciences, University of Warmia and Mazury in Olsztyn, 10-719 Olsztyn, Poland; jerzy.napiorkowski@uwm.edu.pl

**Keywords:** ultrasonic testing, corrosion, longitudinal wave, non-destructive evaluation, elevated temperature

## Abstract

On-stream inspections are the most appropriate method for routine inspections during plant operation without undergoing production downtime. Ultrasonic inspection, one of the on-stream inspection methods, faces challenges when performed at high temperatures exceeding the recommended 52 °C. This study aims to determine the ultrasonic velocity and attenuation with known material grade, thickness, and temperatures by comparing theoretical calculation and experimentation, with temperatures ranging between 30 °C to 250 °C on low-carbon steel, covering most petrochemical equipment material and working conditions. The aim of the theoretical analysis was to obtain Young’s modulus, Poisson’s ratio, and longitudinal velocity at different temperatures. The experiments validated the theoretical results of ultrasonic change due to temperature increase. It was found that the difference between the experiments and theoretical calculation is 3% at maximum. The experimental data of velocity and decibel change from the temperature range provide a reference for the future when dealing with unknown materials information on site that requires a quick corrosion status determination.

## 1. Introduction

As technology advances, today’s petrochemical plants produce more petrochemical derivatives, and therefore such plants have undergone various expansions to meet increased production needs. This expansion also leads to complex plant maintenance, requiring more planning and maintenance time to balance each production line.

Accidents and failures in plants are considered unexpected downtime, and all plant owners strive to avoid them. Turnaround maintenance is one of the methods to shut down plants’ operation for a short period to carry out maintenance work including inspection, repair, alteration, and installation. Because the turnaround requires extensive planning and execution in a limited period of time with several stakeholders, it leads to many considerations such as planning, duration, cost, vendors, policy, potential risk, and suffering from production downtime [1,2].

On-stream inspections are the most appropriate method for routine inspections during plant operation without going through production downtime [3]. Ultrasonic testing to determine material wall loss or corrosion is one of the most commonly used methods in on-stream testing [4,5].

Ultrasonic inspection is widely used in the petrochemical industry, such as in various base materials used in petrochemical equipment; ultrasonic inspection is a more effective way to detect any defective product before leaving the mill to ensure quality. Ultrasonic inspection also plays an important role in the process of construction or expansion of the petrochemical industry; there will be a large number of welds, whether it is welding carried out at the construction site or assembling equipment in the factory, for example, arc welding and friction welding [6], or inspecting the sub-product of the modern manufacturing techniques such as computer numerical control (CNC) machining and injection moulding [7,8].

The most common ultrasonic inspection technique for welds is the pulse-echo A-scan technique [9], which uses a single ultrasound beam probe with a flaw detector to detect the discontinuity via ultrasound reflection signal [10]. Another typical ultrasonic inspection technique used in the field during on-stream inspection to detect material wall loss is ultrasonic thickness gauging (UTG) [11], and there have been some developments in recent years on ultrasonic thickness measurement using unmanned aerial vehicles (UAV) [12,13].

According to Laza [14], many plant owners focus on pressure vessels and heat exchangers but overlook the piping system as a critical asset. A failure or leak in a process piping system can have a significant impact on a business, disrupting production or causing catastrophic consequences in the event of an explosion, fire, or hazardous fluid spill, especially for piping systems that work under high pressure [15]. One of the traditional ways of avoiding leaks in such piping systems is through adequate pre-tensioning of the multi-bolted connections, by which the pipelines are formed [16,17].

Some of the existing plants might have come across equipment and piping systems that require on-stream inspection without information on the material. The ultrasonic velocity calibrated in ambient temperature may show variations at elevated temperatures, causing inaccuracy [18]. The challenges of ultrasonic inspection during plant operation are mainly due to high temperature that exceeds the standard recommended ultrasonic testing temperature of 52 °C, causing erroneous measurements.

Phased array corrosion mapping is an advanced ultrasonic inspection technique that has various advantages over conventional UT (ultrasonic testing) beams. Unlike typical UT beams which only provide information about one thickness at a time, phased array scans can yield many thickness measurements at the same time, leading to improved corrosion detection coverage and higher production speeds [19].

One of the primary advantages of employing a phased array for corrosion mapping is its ability to work similarly to a standard UT probe array. Multiple probes are positioned with perfect overlap and work simultaneously to acquire data in phased array examinations. This enables more thorough and efficient corrosion mapping.

It is crucial to highlight, however, that the use of phased array corrosion mapping at high temperatures is not yet popular. The majority of prior inspections utilising this technique were carried out at room temperature [19,20,21]. Table 1 shows the current working temperatures for phased array corrosion mapping techniques in the NDT (Non-Destructive Testing).

The lack of comprehensive high-temperature phased-array corrosion-mapping studies emphasises the need for additional research and development in this field. As industries and applications require inspections in high-temperature environments, it is critical to investigate the feasibility and effectiveness of employing phased-array techniques for corrosion mapping in such circumstances. This would provide important insights and direction for executing phased-array inspections in high-temperature conditions, resulting in improved corrosion detection and inspection practices.

The objective of this study is to determine the ultrasonic velocity and attenuation with the known material grade, thickness, and temperatures by comparison of theoretical calculation and experiment between 30 °C to 250 °C on low-carbon steel that covers most of petrochemical equipment material and working conditions, thereby facilitating the use of ultrasonic testing in a temperature zone above 52 °C.

The outcome of this study can serve as a reference guide for calibrating ultrasonic equipment that requires working in elevated temperatures, explaining whether ultrasound velocity, attenuation, and other ultrasound properties would require modification to improve the detectability and sensitivity.

A similar approach was used for pre-strained specimens of aluminium alloys at elevated temperatures under monotonic tension and creep condition [25,26].

Some formulae related to the subject of this article will be discussed in the next section to further elaborate and connect with the experimental test.

An actual experimental test on an A36 low-carbon steel plate was carried out via phased-array ultrasonic testing on 30 °C to 250 °C at a step interval of 10 °C. This method was used as phased-array ultrasonic testing has recently become widely recognized due to its better corrosion detection, especially for localized corrosion, and defect sizing accuracy [27].

To imitate plant working conditions, the temperature of the carbon steel plate was raised by utilising heating devices in this investigation. Temperature control accuracy was closely controlled, with a tolerance of ±0.5 °C, utilising thermocouples and a hand-held thermometer. Small-size thermocouples provide accurate temperature measurements [28]. The target temperature range was so obtained and maintained throughout the experiment.

This paper’s discussion and conclusion sections give a thorough examination of the changes found in the ultrasonic characteristics of A36 low-carbon steel as temperature raised. The results show that ultrasonic inspections on this material up to 250 °C are possible. However, it is critical to evaluate the trade-off between altering the decibels (dB) and the worries about velocity variations.

According to this study, as the temperature rose, there was an increase in decibels (dB), indicating a higher level of attenuation. Because of characteristics such as absorption and diffraction, this is an unavoidable phenomenon in ultrasonic testing. The dB adjustment trade-off is required to assure the inspection’s detection sensitivity and reliability.

Furthermore, the study emphasises the concern about changes in velocity as temperature varies. It is critical to consider how temperature affects the longitudinal velocity of the ultrasonic wave. To achieve precise and reliable measurements during ultrasonic examinations, the study emphasises the importance of rigorous analysis and comprehension of these changes.

In this study, the theoretical calculation results of the change of ultrasonic properties caused by an increase in temperature have been proven through practical experiments. It has been found that between the experiments and theoretical calculation, it is 3% at maximum, as listed in detailed longitudinal velocity.

Professionals can efficiently evaluate the condition of materials in real-time settings by leveraging experimental data in conjunction through the use of a spectrometer and theoretical computations. This technique enables them to make informed corrosion assessment decisions, ensuring the integrity and dependability of structures and equipment in a variety of working situations.

More specifically, in future experiments, which could include complex specimens of different depths, it is possible to use the MCAD system to design a 3D model and send it to the specimen for fabrication [29,30].

## 2. Theory and Methodology

This section introduces some related ultrasound equations used to verify the experimental test. First, the ultrasound longitudinal velocity was obtained via calculation using A36 carbon steel Young’s modulus and Poisson’s ratio. On another hand, the same ultrasound longitudinal velocity was obtained from the actual experiment. Second, the velocity can be further calculated via temperature coefficients added every 10 °C from 30 °C to 250 °C. A similarly experiment was also conducted at every 10 °C step up to 250 °C as well as for ultrasonic attenuation. This section describes the methodology and setup used to experimentally determine ultrasound velocity and attenuation caused by temperature rise on carbon steel.

### 2.1. Ultrasonic Attributes

The typical sound waves for ultrasonic testing are longitudinal, share, and Lamb waves. A longitudinal wave, also known as a straight beam, was used to detect material loss, internal lamination, or corrosion. Ultrasonic thickness gauging, pulse-echo A-scan ultrasonic testing, and phased-array ultrasonic testing utilise the longitudinal wave.

Longitudinal waves are compressional waves that propagate in a medium by making the particles parallel to the wave’s propagation direction. In other words, the particles oscillate back and forth along the wave’s axis. This sort of wave motion produces alternating zones of compression and rarefaction, resulting in high-and-low pressure regions.

Because of their capacity to penetrate deep into materials and provide useful information about their internal condition, longitudinal waves are often used in ultrasonic testing. One of the most common applications of longitudinal waves is ultrasonic thickness gauging, which involves sending a wave through a material and measuring the time it takes for the wave to travel through the material and reflect back. This approach is frequently used in a variety of sectors to evaluate the structural integrity of pipelines, tanks, and other structural components.

Longitudinal waves are essential in ultrasonic testing because they provide a reliable and effective method of detecting material loss, lamination, and corrosion. Longitudinal waves are excellent for a wide range of applications due to their ability to penetrate deep into materials and provide vital information about their interior state. Understanding the features and applications of longitudinal waves allows ultrasonic testing specialists to optimise their inspection processes and provide reliable structural integrity assessments in a variety of sectors.

The first equation represents the basic link between frequency (f), wavelength (λ), and phase velocity (v). It describes the interaction of these three characteristics for an ultrasonic wave travelling through a material.

The phase velocity of an ultrasonic wave is directly proportional to its frequency and wavelength, according to this equation. The phase velocity is the rate at which the ultrasonic wavefronts propagate across the medium.

This equation can be used to derive a variety of helpful relationships. If the frequency and wavelength are known, for example, the phase velocity can be determined. If the phase velocity and frequency are known, the wavelength can be calculated. This equation is crucial in understanding the behaviour of ultrasonic waves and their interactions with various materials. It is widely utilised in ultrasonic testing and other applications where the propagation characteristics of ultrasound waves must be analysed and comprehended.

(1) V=fλ

where: V = velocity; f = frequency; λ = wavelength

(2) V=x/t

Equation (2) provides an alternative method for estimating ultrasonic velocity in addition to the previously discussed equation. This equation connects the velocity of an ultrasonic wave to the time it takes the wave to traverse a certain distance through a material. The distance travelled, indicated as (x), reflects the round-trip distance of the signal via the sample and back.

To compute the velocity using Equation (2), first, determine how long it takes the ultrasound signal to travel the round-trip distance. This is accomplished by calculating the time delay between successive echoes received from the substance. The interval between echoes is equal to twice the sample thickness.

The velocity of the ultrasonic wave can be properly estimated by applying Equation (2) and measuring the essential parameters, such as round-trip distance and duration of travel.

Equation (2) establishes a key relationship that enables quantitative measurement of ultrasonic wave propagation within materials. Using this equation and exact measurements, practitioners can extract significant information about the material’s properties, allowing for the optimisation of ultrasonic testing techniques as well as the enhancement of detectability and sensitivity in a variety of applications.

Ultrasonic velocity measurements can be used to derive parameters such as Young’s modulus, Poisson’s ratio, acoustic impedance, and other factors when the density of a solid material is known.

The modulus of elasticity, Poisson’s ratio, and material density all play important roles in determining how fast bulk waves propagate in a material. Equations (3) and (4), as referred to in [31], give mathematical formulations for computing longitudinal and shear wave velocities, respectively. As input parameters, these equations use the modulus of elasticity, Poisson’s ratio, and density values.

It is now possible to estimate and analyse crucial material properties by properly monitoring ultrasonic velocities and using the necessary equations. This data can be extremely useful in a variety of domains, including materials science, engineering, and non-destructive testing, allowing for greater knowledge of material behaviour and facilitating informed decision making in practical applications [32].

(3) $$C_{longitudinal}=\sqrt{\frac{E\;\left(1-v\right)}{\rho \left(1+v\right)\left(1-2\sigma v\right)}}$$

(4) $$C_{transverse}=\sqrt{\frac{E\;}{2\rho \left(1+v\right)}}=\sqrt{\frac{G}{\rho }}$$

where: v = Poisson’s ratio, *ρ* = material density, G = Shear modulus, E = Young’s modulus $C_{longitudinal}$ = longitudinal velocity, $C_{transverse}$ = transverse (shear) velocity [33].

Poisson’s ratio is known as transverse contraction per unit width divided by longitudinal extension per unit length under simple stress. The Poisson’s ratio can also be calculated when the longitudinal and share velocities are known, as described in Equation (5) [34]. In Equation (6), determining Young’s modulus requires material density and sound velocity [35].

(5) $$v=\frac{\left[1-2{\left(\frac{V_{s}}{V_{l}}\right)}^{2}\right]}{2\left[1-{\left(\frac{V_{s}}{V_{l}}\right)}^{2}\right]}$$

(6) $$E=\frac{\left[{\rho V_{s}}^{2}(3{V_{l}}^{2}-{4V_{s}}^{2})\right]}{{V_{l}}^{2}-{V_{s}}^{2}}$$

where: v = Poisson’s ratio, *ρ* = material density, G = Shear modulus, E = Young’s modulus of elasticity V_l_ = longitudinal velocity, V_s_ = transverse (shear) velocity [33].

The phenomenon of ultrasonic attenuation happens during the propagation of sound waves and is impacted by factors such as absorption and diffraction. As a result of these circumstances, energy is lost when the sound wave travels and reflects back. Analysing the attenuation record, which calculates the attenuation in decibels (dB) based on the first two echoes of the data signal, yields the overall attenuation of the sound wave [36]. The energy loss per unit length, reported in decibels per metre, is frequently used to quantify attenuation. This parameter provides useful information regarding the rate at which the energy of a sound wave decreases as it travels through a medium.

Understanding and characterising ultrasonic wave attenuation is critical in a variety of applications, including Non-Destructive Testing and material evaluation. The attenuation factor α can be calculated using Equation (7), where V1 is the amplitude without loss, and V2 is the amplitude with loss. The attenuation dB can also be obtained by increasing or reducing it until it reaches the reference level, such as 80% FSH by particle [37].

(7) $$\alpha =20\;log\frac{V1}{V2}$$

In addition to the above-listed attributes, ultrasonic velocity measurements can provide information about the following material characteristics:

1. Grain Size: The grain size of polycrystalline materials can be estimated via ultrasonic velocity measurements. The interaction between ultrasonic waves and grain boundaries influences the velocity of the waves, allowing the average grain size of the material to be calculated [38].
2. Phase Transformations: Under certain temperature or pressure circumstances, some materials undergo phase transformations, such as solid–solid or solid–liquid transitions. Ultrasonic velocity measurements can detect these phase transitions by detecting variations in velocity as the material transforms, making phase diagrams and material behaviour easier to investigate [39].
3. Analysis of Stress and Strain: Ultrasonic velocity data can be used to evaluate the effects of stress and strain on the material. Variations in velocity can reflect changes in the mechanical characteristics of a material, providing insight into the presence of residual stresses or deformation mechanisms [40].
4. Elastic Anisotropy: Elastic anisotropy refers to the directional dependency of certain materials’ elastic characteristics. Ultrasonic velocity measurements can help characterise and quantify this anisotropy, providing vital information on the mechanical behaviour of the material in different directions [41].

This paper aims to determine the theoretical calculation of velocity change and attenuation when the temperature rises to 250 °C. Hence, the following formulae are essential to calculate ultrasound attributes with temperature coefficients added [42].

(8) $$M\left(T\right)=M\left(T_{0}\right)+\frac{\partial M(T)}{\partial T}\;∆T$$

Equation (8) gives a broad calculation not limited to sound velocity but to other ultrasound attributes [43]. Where M is one of the sample’s mechanical properties, such as Young’s modulus, Shear modulus, Poisson’s ratio, and bulk modulus; $M\left(T\right)$ is the ultrasound attribute in solids at a given temperature, $M\left(T_{0}\right)$ is the reference temperatures, ∆T= solids’ temperature change, and $\frac{\partial M(T)}{\partial T}$ is the temperature dependence coefficient. The $\frac{\partial M(T)}{\partial T}$ reported by many researchers for Young’s modulus is −0.0803 GPa/°C, and for Poisson’s ratio, it is 2.4 × 10^−5^/°C [33]. The theoretical calculation in this paper was first to obtain Young’s modulus and Poisson’s ratio in different temperatures using Equation (8), and after that, use Equation (3) to calculate longitudinal velocity in different temperatures.

### 2.2. Experiment Test

The experiment was conducted on the phased array ultrasonic testing equipment with a 5LA12-0-L 5 MHz probe and a high-temperature wedge. The selected test specimen was a 200 mm × 200 mm × 25 mm carbon steel plate. Portable spectroscopy was carried out to verify the carbon steel material grade and obtain the material’s chemical properties, as shown in Table 2. The chemical properties matched the carbon steel listed in ASME II—Table TM-1 and Table PRD.

The ASME II—Table TM-1 given the carbon steels with carbon content < 0.30% material grade Young’s modulus is 201.73 GPa in ambient temperature (30 °C) with interpolation calculation, ASME II—Table PRD shows the material density for carbon steels is 7750 Kg/m^3^, and Poisson’s ratio is 0.3. Therefore, the theoretical longitudinal velocity and shear velocity can obtained via Equation (3) and Equation (4), respectively.

The test specimen was first tested at ambient temperature; it used the principle of Equation (2) to measure two back wall echoes (round trip) via pulse-echo A-Scan with known thickness. It shows that the longitudinal velocity by experiment is 5915.8 m/s, the calculated longitudinal velocity is 5919.5 m/s at 30 °C, and the difference is 0.06%.

Additional parameters such as density values, Young’s modulus, and Poisson’s ratio were calculated for the temperature range of 30 °C to 250 °C to validate the accuracy of the theoretical calculations. To determine these characteristics, the thermal coefficient was added to Equation (8), which took into consideration the material’s thermal expansion.

In addition, Equation (3) was utilised to compute the theoretical longitudinal velocity at various temperatures. Based on the material parameters and temperature, this equation may estimate the longitudinal wave velocity.

**Figure 1:**
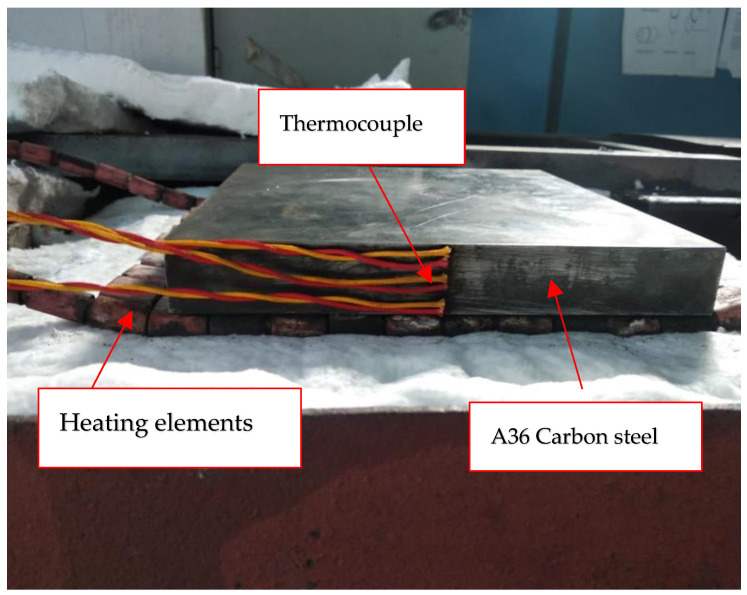
Experiment setup on A36 carbon steel material.

The test specimen was put on a heating element in the experimental setup, as shown in Figure 1. Three thermocouples were strategically placed on the top, centre, and bottom of the material to measure the temperature increase. At each phase, the temperature was allowed to stabilise for at least 25 min, ensuring an accuracy of 0.5 °C.

The precise back wall approach was used for the experimental measurements, which involved measuring the time it takes for the ultrasonic wave to travel through the specimen and reflect back from the opposite surface. The temperature was gradually raised from 30 °C to 250 °C in 10 °C increments.

The authors hoped to test the accuracy and dependability of the theoretical models and expand their understanding of ultrasonic wave behaviour in changing temperatures by undertaking these experiments and comparing the measured results with the theoretical calculations.

The experiment was performed after the temperature verification via thermocouples and a hand-held thermos meter in each step to record the ultrasound longitudinal velocity at the temperature change.

The amplitude of the sound echo might decrease as the temperature rises; this process is known as ultrasonic attenuation. To measure and record the attenuation, a reference point is established at ambient temperature by establishing the echo amplitude as the baseline or reference level at 80% of the full-screen height (FSH) [44].

The echo amplitude tends to decrease as temperature rises due to a variety of variables such as increased energy absorption and scattering within the material. To effectively evaluate ultrasonic attenuation, the echo amplitude is increased to maintain it at 80% FSH during each succeeding test at higher temperatures. The difference in dB measurements from the reference level offers useful information regarding the attenuation of the ultrasonic signal as it changes with temperature.

Researchers and engineers can determine the level of attenuation induced by temperature differences by monitoring and analysing dB changes. This data is critical for understanding the behaviour of materials at various temperatures and can be used to optimise ultrasonic testing methodologies and equipment settings for accurate fault discovery and evaluation.

The advantage of this method is that when a rapid corrosion state diagnosis of an unknown material is required in the field, the machine can be set up quickly by examining the composition of the material and applying calculations to obtain a value for velocity. The data from this experiment can also be used for future applications, e.g., using finite element analysis [15,45].

## 3. Results and Discussion

**Figure 2:**
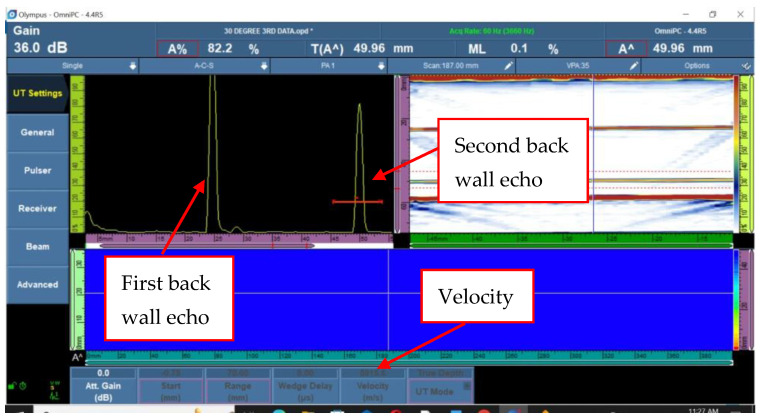
Ultrasonic velocity and decibels by experiment.

The experiment PAUT data in Figure 2 indicate the material’s first and second back wall echo with a known material thickness of 25 mm. The equipment can obtain the longitudinal velocity when the two back wall echoes were set at a distance of 25 mm and 50 mm, respectively. To calculate the longitudinal velocity, the back wall echo amplitudes are set to 80% of the FSH. This amplitude modification gives a consistent reference level for comparison. The related dB values are recorded for further analysis and comparison. The velocity of the material can be calculated by analysing the dB values obtained from back wall echoes at various distances. This velocity information is useful for analysing the material’s characteristics and detecting any potential imperfections or defects.

**Figure 3:**
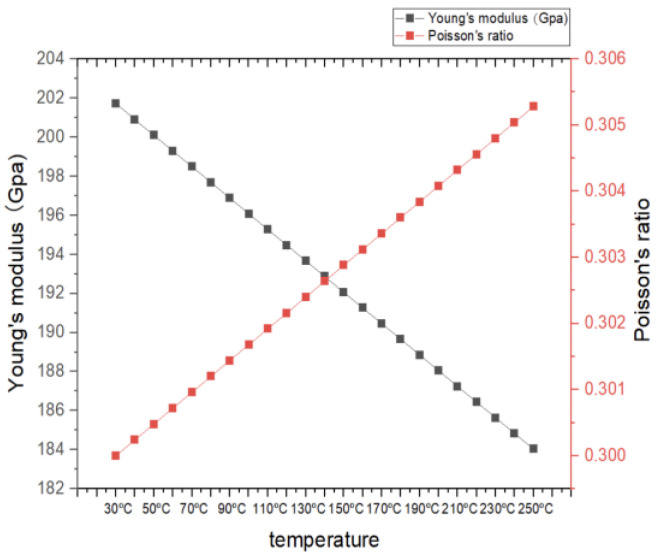
Theoretical calculation of Young’s modulus and Poisson’s ratio.

Figure 3 shows the plot chart of Young’s modulus and Poisson’s ratio from 30 °C to 250 °C calculated via Equation (8). It indicates that Young’s modulus decreases consistently when the temperature increases and Poisson’s ratio increases when the temperature increases, similar to the findings of other researchers [33].

Three sets of data were taken at each temperature to record the longitudinal velocity in the temperature range of 30 °C to 250 °C. It was critical to provide enough time between tests 1 and 3 for the material to attain the correct temperature range. The tests were first carried out in a continuous manner, but when the temperature went over 100 °C, the variance between the three sets of measured velocity increased.

The cooling effect of the couplant employed as a medium for the studies can explain this variation. When the experiments were run in series, the couplant from the first experiment cooled the surface of the specimen, impacting the succeeding trials. As a result of the cumulative cooling effect, the surface in the third experiment, which was the last in the sequence, would be the coldest.

**Figure 4:**
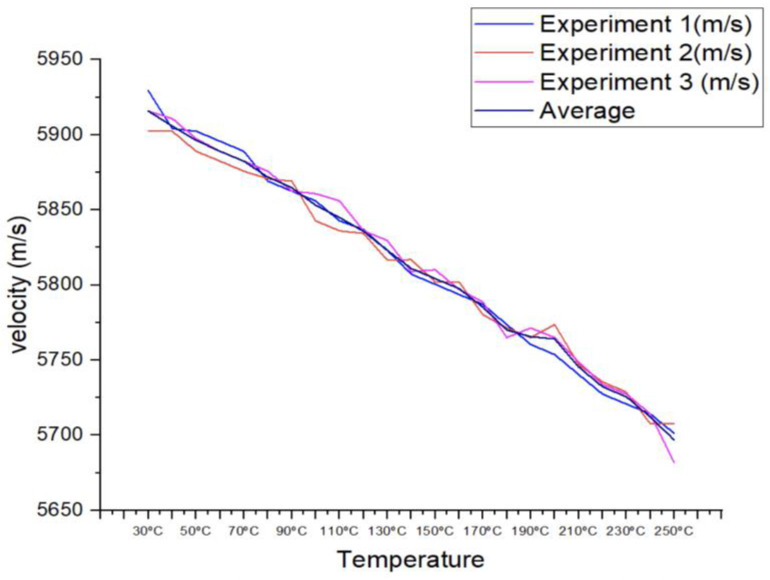
Summary diagram of ultrasonic velocity change experiment.

The mitigation action should allow enough time for the material to rise in temperature between experiments 1, 2, and 3. It must be ensured by using a thermos meter before carrying out the test. This action can be seen as the initial form of the technical process description, which will later turn into a more rational form [46]. Three sets of obtained longitudinal velocities and average velocities are shown in Figure 4.

The longitudinal velocity data produced from this study might give vital insights into the knowledge of how temperature influences the ultrasonic characteristics of the tested material by carefully regulating the experimental settings and accounting for elements that may induce variability.

**Figure 5:**
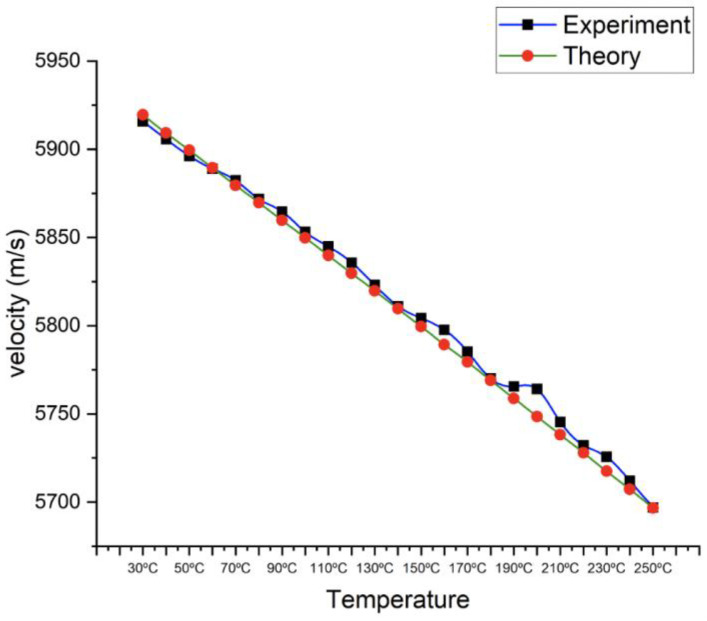
Comparison between theoretical calculation and experimental determination of ultrasonic velocity change.

Figure 5 compares the longitudinal velocity by experiment with the calculated longitudinal velocity from 30 °C to 250 °C. The averaged experiment data show that the longitudinal velocity is 5915.83 m/s at ambient temperature, and when the temperature increases to 250 °C, the longitudinal velocity gradually decreases to 5696.9 m/s. On the other hand, the calculated longitudinal velocity starts at 5919.5 m/s and decreases to 5696.7 m/s when linearly decreasing using the calculated Young’s modulus and Poisson’s ratio data. Table 3 shows both longitudinal velocity differences from 30 °C to 250 °C, and the variation does not exceed 3%.

**Figure 6:**
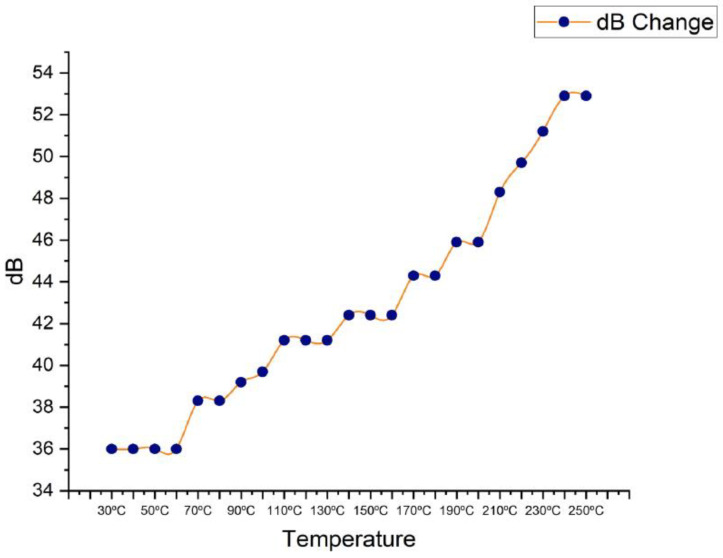
Decibels (dB) change between 30 °C to 250 °C.

The decibels were measured during the temperature rise experiment by settting the first back wall at 80% of full-screen height (FSH). This decibel measurement was saved for future use. The data demonstrated that when the temperature grew, so did the decibel level. Interestingly, as shown in Figure 6, the three experiments conducted in this study consistently indicated an increase in decibels beginning at 36 dB at 30 °C and progressing to 52.9 dB at 250 °C.

These findings contrast slightly from those of another researcher, who attributed attenuation variations to differences in material grain structure or grain size [47]. They do, however, agree with the findings of another study, which found a consistent increase in attenuation with higher temperatures using a different approach [48].

These comparisons show the need for considering numerous elements when interpreting and analysing ultrasonic attenuation data, such as material qualities and testing procedures. The continuous increase in decibels seen in this study adds to the body of knowledge on this subject by providing useful insights into the relationship between temperature and ultrasonic attenuation.

In summary, when the temperature is from 30 °C to 250 °C, the longitudinal velocity gradually decreases, the experiments data mostly match with the calculated longitudinal velocity, but on the other hand show increasing in decibels due to high attenuation

## 4. Conclusions

In this study, the theoretical calculation results of the change in ultrasonic properties caused by an increase in temperature have been proven through practical experiments. It was found that the difference between the experiments and theoretical calculation is 3% at maximum, as listed in detail as longitudinal velocity in Table 3.

Base metals and operating temperatures typically differ between job locations. The experimental data obtained in this study from the temperature range serve as a significant reference for future applications when quick corrosion status diagnosis for unknown materials on site is necessary, for example, using FEM. By comparing the collected velocity and decibel change data to the reference values, it is possible to quickly analyse a material’s condition.

A spectrometer can be used to validate the chemical composition of the material and establish the working temperature of ultrasonic equipment to speed up the calibration process. Once these values have been determined, the ultrasonic velocity can be estimated theoretically. This method not only saves significant resources but also improves work efficiency by removing the need for rigorous testing and measurement.

Professionals can efficiently evaluate the condition of materials in real-time settings by leveraging experimental data in conjunction with the use of a spectrometer and theoretical computations. This technique enables them to make informed corrosion assessment decisions, ensuring the integrity and dependability of structures and equipment in a variety of working situations.

## Figures and Tables

**Table 1:** The existing operating temperatures used in phased array corrosion mapping.

Company	Temperature Range	Reference
RollerFORM scanner	25–80 °C	[22,23]
RMS SCUT/Dual element	25–200 °C	
SyncScan 2	−10–45 °C	[24]

**Table 2:** Tested specimen’s material chemical composition.

Element	Carbon (C)	Silicon (Si)	Iron (Fe)	Phosphorus (P)	Sulfur (S)
Content	0.146%	0.275%	97.79%	0.018%	0.021%

**Table 3:** Longitudinal velocity between experiment and calculation.

Temperature	Experiment (m/s)	Calculated (m/s)	Temperature	Experiment (m/s)	Calculated (m/s)
30 °C	5915.8	5919.5	150 °C	5804.2	5799.5
40 °C	5905.7	5909.2	160 °C	5797.6	5789.3
50 °C	5896.2	5899.4	170 °C	5785.3	5779.5
60 °C	5889	5889.5	180 °C	5770	5769
70 °C	5882.3	5879.6	190 °C	5765.6	5758.8
80 °C	5871.8	5869.7	200 °C	5764.1	5748.5
90 °C	5864.7	5859.7	210 °C	5745.4	5738.2
100 °C	5853.1	5849.8	220 °C	5732.3	5727.9
110 °C	5844.9	5839.8	230 °C	5725.6	5717.5
120 °C	5835.7	5829.7	240 °C	5712.1	5707.2
130 °C	5823.1	5819.7	250 °C	5696.9	5696.7
140 °C	5811	5809.6			

## Data Availability

All data generated or analysed during this study are included in this article.
